# Belt and road initiative and healthy silk road: an alternative path for Pacific island countries to participate in global public health governance

**DOI:** 10.1186/s41256-023-00329-8

**Published:** 2023-10-19

**Authors:** Yujie Mei

**Affiliations:** 1 School of Marxism, Wuhan University of Science and Technology, Wuhan, 430081 Hubei China; 2 China Institute of Boundary and Ocean Studies, Wuhan University, Wuhan, Hubei China

**Keywords:** The belt and road initiative, Pacific island countries, Public health, COVID-19

## Abstract

Pacific island countries (PICs) located in a region with relatively insufficient capacity to respond to public health emergencies, establishing reliable public health cooperation is conducive to maintaining security and stability. The belt and road initiative (BRI) launched by China attempts to provide a novel form of international cooperation and has supported multi-channel investment and construction. This article elucidates the history of public health cooperation between China and PICs, as well as the current situation of the BRI in the field of public health and emphasizes that there are numerous constraints in the public health cooperation between China and PICs. Given the profound impact of COVID-19 on diplomatic strategies, gradual cooperation in the field of public health may be the initial exploration of the BRI in the PICs, but it also means that the initiative must deal with challenges from geopolitical competition and cultural differences.

## Introduction

With the rapid deepening of international exchanges, health problems are no longer simply issues for a single nation [1]. We have previously pointed out that the crisis of COVID-19 is calling on Pacific island countries (PICs) to prepare ahead of time [2]. Gratefully, the pandemic seems to be effectively preserved after strict controls have been implemented in several PICs [3], but it has also brought a significant backward impact on the economic and social prospects for further development of this region. PICs are characterized by small populations (only 6.6 million) with a large proportion of the population engaged in subsistence agriculture. Four countries of PICs are classified as least developed countries (Kiribati, Samoa, Solomon Islands, Tuvalu, and Vanuatu), the rest are listed as developing countries. Economic conditions in the region are not encouraging, with some countries experiencing annual gross domestic product (GDP) growth fall by 3% over the past year [4]. Remote locations, high communication and transportation costs, fragile environment, and inadequate infrastructure present public health governance dilemmas. Therefore, the establishment of a long-term effective public health and infectious disease surveillance system is extremely urgent for PICs. China has been committing to integrating public health into its economic cooperation, participating in the BRI is beneficial to improving public health governance capacity, but potential risks such as geopolitical and cultural differences are worth considering.

## The healthy silk road

The BRI was officially launched by the Chinese government as an open arrangement to promote trade and expand international financial cooperation in 2013. As of August 2022, China has engaged 149 countries and 32 international organizations in the BRI, containing 70% of the world’s population, and 30% of the global GDP [5]. Scarred by the epidemic of Severe Acute Respiratory Syndrome (SARS) in 2003 when trade was paralyzed and economic loss was enormous, the Chinese government aware of the interconnection of economy and health, trying to increase its global leadership in the field of public health through the BRI, and put forward the healthy silk road (HSR) in 2017 [6]. One of the core goals of the BRI is to enhance global cooperation and increase investment in public health, aiming to provide a shared platform to strengthen and upgrade the capacity to handle regional public health emergencies through the sharing of epidemiological information, the exchange of prevention and intervention methods, and the training of skilled health professionals.

The BRI calls for a development model that combines bilateral and multilateral cooperation. For a long time, China’s bilateral public health diplomacy has been mainly conducted with other developing countries. With the promotion of multilateral cooperation actions in the field of health, increasing bilateral cooperation and assistance projects are carried out under the framework of the multilateral global health governance system with World Health Organization (WHO) and the United Nations (UN) as the core. In addition to participating in the global health governance framework dominated and led by the WHO, China has established multilateral health partnerships with most countries and international organizations along the BRI: Trans-regional multilateral mechanisms (such as the China-Central, and Eastern European Countries Health Ministers Forum), regional multilateral mechanisms (such as the China-Association of Southeast Asian Nations Forum on Health Cooperation). Some positive results have been achieved, such as leading the establishment of the BRI International Medical Education Alliance (BRIMEA) and helping to establish the African Centers for Disease Control and Prevention (CDC). Since the pandemic of COVID-19, several BRI partner countries have taken advantage of the HSR platform to regularly share information and exchange experiences on COVID-19 prevention through virtual conferences, webinars, and conferences. In addition, China has provided medical assistance to partners, and has started to donate vaccines to some low- and middle-income countries. Even though most of this medical diplomacy was wrapped within the framework of HSR, however, with the expansion and deepening construction of the BRI, especially considering the adverse impact of the pandemic of COVID-19, the progress of cross-regional cooperation presents new challenges.

## Public health cooperation between China and PICs

The Western Pacific is the key region for China to propose economic cooperation through the BRI, public health governance is a critical factor that affects the harmonious development of the economy and society in this region [7]. Traditionally, this region is part of the Western sphere of influence, but some PICs have begun to seek benefit from the rivalries between the major powers. Fiji and Samoa are the first two PICs to establish diplomatic relations with China, with a positive attitude towards the BRI. However, most PICs hold a cautious wait-and-see attitude. Therefore, the previous public health cooperation between China and PICs was mainly based on medical assistance and multi-form cooperation and exchanges between the authorities (Table 1). After the in-depth implementation of the BRI, China’s National Health Commission launched the BRI-supported three-year implementation program for health exchange and cooperation in 2015, which defined new cooperation mechanisms and extend the scope of bilateral and multilateral cooperation to the prevention and control of infectious diseases, training of public health personnel, traditional medicine, reform of the health system and policy, and development of the health industry, further enriches the public health partnership model. Since 2018, 10 island countries in this region have signed agreements with China to jointly build the BRI. Some achievements have been made, such as the aid construction of medical institutions (Samoa National Medical Center, Fiji NAVUA Hospital), the regular dispatch of medical personnel and material donations. Furthermore, the BRIMEA was an academic organization established in 2018, and currently has 86 medical education institutions from 23 countries as members. With the support of the BRIMEA, medical schools, healthcare institutions, and medical talents in PICs can get more opportunities for international exchanges and cooperation. Scientific research and exchanges with the members [8]. In addition, health tourism is also one of the main areas of cooperation between countries along the BRI around the big health industry, PICs have rich tourism resources for investment and development. It is foreseeable that under the BRI framework, in addition to providing medical assistance, cooperation in medical services, such as equipment and infrastructure construction, medical education, scientific research, loans, and financial input, will be further strengthened. In other words, the public health cooperation between China and PICs is most likely developing from a single field of health cooperation to diversified cooperation.

**Table 1:** Signed cooperation documents on public health between PICs and China

Year	Country/Territory	Signed documents	Scopes
1993	Vanuatu	Agreement on China sending a medical team to Work in Vanuatu	Dispatch medical team
1996	Marshall	Agreement on China’s dispatch of a medical team to Marshall	Dispatch medical team
2002	Papua New Guinea	Agreement on The Dispatch of Chinese Medical Teams to Work in Papua New Guinea	Dispatch medical team
2002	Fiji	Agreement on the Employment of Chinese Doctors by the Government of Fiji	Fiji's government employs Chinese medical personnel
2003	Vanuatu	Cooperation Agreement on Animal quarantine and Animal Health	To prevent the cross-border spread of animal-borne infectious diseases
2003	Vanuatu	Cooperation Agreement on Plant quarantine	Prevent the spread of organisms harmful to plants and plant products
2003	New Zealand	Memorandum of Understanding on Cooperation in Food Safety Science and Technology	Scientific and technological cooperation in the field of food safety and food standards
2005	Papua New Guinea	Memorandum of Understanding on Entry-exit animal and Plant Quarantine and Food Safety Cooperation	Measures for the administration of entry and exit of animals and plants
2006	Papua New Guinea	Agreement on China sending a medical team to Work in Papua New Guinea	Dispatch medical team
2007	New Zealand	Agreement on health Cooperation	To jointly improve public health undertakings and to promote exchanges between medical institutions and personnel

Like most countries, the motives for foreign aid are complex, the difference is that as an emerging economy, China now leans more toward commerce and trade and has relatively limited partners, and simple cooperation models and the progress is still in its early stage. Since the outbreak of COVID-19, China has carried out numerous medical and health cooperation with PICs, such as video dialogue meetings between leaders and experts, health medical supplies, and product donators [9]. The Chinese government chooses this region to carry out frequent actions to establish a responsible image of emerging international governance and play a beneficial role in promoting its BRI in other regions and countries. Given the historical legacy of colonialism in the PICs, the U.S. government and its allies have placed a premium on maintaining dominance in this region. The U.S. has already begun re-engaging economically in the Pacific. In 2022, Fiji, one of China’s significant partners in PICs, became the first PIC to join President Biden’s Indo-Pacific Economic Framework (IPEF). In addition, risks of political changes, debt repayment ability, concerns about the transparency of Chinese companies, and concerns about increasing regional imbalances and geopolitical competition are among the topics discussed around the BRI [10]. But it is worth noting that, the presence of BRI has provided an alternative to continued dependence on ex-colonial powers, providing these countries with “greater bargaining power”, enabling them to diversify their sources of aid and support, as well as provide more development opportunities for public health governance.

## Conclusions

The COVID-19 pandemic has exposed the shortcomings and weaknesses in global public health governance. The BRI promotes investment in the medical and health sector, boosts pharmaceutical trade and personnel exchanges, and is conducive to accelerating the construction of medical and health systems in countries along the route and continuously improving the capacity of public health services. Therefore, the field of public health is a vital breakthrough and direction for the cooperation between China and the PICs to jointly build the BRI, as well as an alternative path for PICs to participate in global public health governance. However, the fragile environment, the negative impacts of climate change, and the relatively weak public health infrastructure and medical care elevate the complexity of achieving health security and building modern health systems in PICs. In addition, due to factors such as geopolitics, debt risks, and local acceptance of the BRI, the expansion of the influence of the BRI in the field of public health cooperation faces significant challenges.

## Data Availability

Not applicable.

## Abbreviations

COVID-19: Corona Virus Disease 2019
PICs: Pacific island countries
BRI: Belt and road initiative
BRIMEA: BRI International Medical Education Alliance
GDP: Gross domestic product
HSR: Healthy silk road
IPEF: Indo-Pacific economic framework
